# Economic evaluation of non-pharmacological interventions in Alzheimer's disease

**DOI:** 10.1590/1980-5764-DN-2024-0219

**Published:** 2025-06-02

**Authors:** Margarida Sobral, Susana Oliveira

**Affiliations:** 1 Universidade do Porto, Health Research and Innovation (RISE-Health), Instituto de Ciências Biomédicas de Abel Salazar, Porto, Portugal.; 2 Unidade Local de Saúde de Santo António, Hospital de Magalhães Lemos, Serviço de Psicogeriatria, Porto, Portugal.; 3 Universidade do Porto, Faculdade de Economia, Porto, Portugal.

**Keywords:** Alzheimer Disease, Healthcare Models, Quality of Life, Quality-Adjusted Life Years, Cost-Benefit Analysis, Doença de Alzheimer, Modelos de Assistência à Saúde, Qualidade de Vida, Anos de Vida Ajustados por Qualidade de Vida, Análise Custo-Benefício

## Abstract

Alzheimer's disease (AD) has significant physical, psychological, and socioeconomic impacts, shortens life expectancy, and reduces quality of life. Non-pharmacological interventions (NPIs) in dementia provide health gains.

**Objective:**

The aim of this study was to conduct a cost-utility study of NPIs in AD, assessing both the costs and health gains associated with cognitive stimulation interventions in AD patients.

**Methods:**

A sample of 40 patients undergoing NPIs and another 40 individuals (control group) were included. Data collected included sociodemographic, clinical, cognitive, and functional information, as well as health status, quality of life, and outpatient costs.

**Results:**

The NPI, considering the discounted cost value of €21,621.31 and the discounted quality-adjusted life year (QALY) gain of 0.81333, resulted in an estimated cost per QALY of €26,583.76. This cost per QALY is within the threshold generally considered acceptable by regulatory authorities in Portugal and in several European countries.

**Conclusion:**

This study supports the recommendation that interventions adjusted to the needs of patients with AD should be implemented, which may include NPIs providing both health gains and economic value.

## RESUMO.

A doença de Alzheimer (DA) tem impactos físicos, psicológicos e socioeconômicos significativos e diminui a esperança e a qualidade de vida. As intervenções não farmacológicas na demência proporcionam ganhos em saúde.

**Objetivo:**

Realizar um estudo de custo-utilidade das intervenções não farmacológicas na DA, avaliando tanto os custos como os ganhos em saúde associados às intervenções de estimulação cognitiva em doentes com DA.

**Métodos:**

Analisaram-se uma amostra de 40 pacientes submetidos à intervenção não farmacológica e outra de 40 pacientes do grupo controle. Os dados recolhidos incluíram informação sociodemográfica, clínica, cognitiva e funcional, bem como o estado de saúde, a qualidade de vida e os custos ambulatoriais.

**Resultados:**

A intervenção não farmacológica, considerando os valores de custos descontados de € 21.621,31 e os ganhos de anos de vida ajustados por qualidade de vida (QALY) descontados de € 0,81333, resultou num custo estimado por QALY de € 26.583,76. Esse custo por QALY se encontra em conformidade com o limite geralmente considerado aceitável pelas autoridades reguladoras em Portugal e em vários países europeus.

**Conclusão:**

Este estudo suporta a recomendação de que sejam implementadas intervenções ajustadas às necessidades dos pacientes com DA, nomeadamente intervenções não farmacológicas, que proporcionem ganhos em saúde e valor econômico.

## INTRODUCTION

Dementia is a syndrome in which there is a deterioration in cognitive and functional abilities, leading to the loss of people's autonomy. This disease has a physical, psychological, social, and economic impact, not only on people with dementia (PWD) but also on their caregivers, families, and society in general. The incidence and prevalence of dementia increase exponentially with age beginning at 65 years^1,2^. Alzheimer's disease (AD), the most common cause of dementia, is a clinical syndrome caused by neurodegeneration, and is characterized by a progressive deterioration in cognitive capacity and the ability to live independently^3^.

Dementia has a huge economic impact^4^ and in the long term, it will be one of the most expensive diseases for society^5^. Prince et al.^6^ estimated that there were 35.6 million PWD worldwide in 2010, and it is expected to increase to 65.7 million in 2030 and 115.4 million in 2050. According to Wimo et al.^7^, the total costs of dementia in the European Union, across 27 countries, were €177 billion; while in northern Europe the direct costs were considerable, in southern Europe, the cost of informal care is the main component of costs.

In the United States, the costs of health care and long-term care for individuals with AD or other dementias are substantial, and dementia is one of the costliest conditions to society^1^, with total payments in 2024 for all individuals with AD or other dementias estimated at US $360 billion. In the United Kingdom, dementia is estimated to have the lowest healthcare costs among four diseases (£1.2 billion, compared with £4 billion for cancer, £2.2 billion for coronary heart disease, and £1.6 billion for stroke) but significantly higher social care costs^8^. A Spanish study concluded that of 19 brain disorders, dementia was the costliest, given its relatively high prevalence rate^9^.

Dementia shortens life and reduces the quality of life (QOL) of people with the disease, with these impacts being difficult to measure and quantify. Dementia, namely AD, imposes high costs on health systems and leads to more hospitalizations per year compared to individuals of the same age without dementia. Health services for other serious medical conditions are strongly affected by the presence of dementia^1^.

Anti-dementia drugs and disease-modifying therapies developed to date have limited effectiveness, although numerous new treatments are being investigated^10^. Several studies highlight the importance of combining pharmacological interventions (PIs) and non-pharmacological interventions (NPIs) as the ideal methodology to obtain the best results during the evolution of dementia^11–13^. Alternative or complementary interventions to PIs are implemented due to the need to maintain or improve cognitive functioning, performance in activities of daily living, and, in general, the QOL of PWD and their caregivers^14^.

NPIs, namely cognitive stimulation (CS), refer to a set of interventions that aim to maximize the person's cognitive functioning and well-being, as well as help them in the process of adapting to the disease. Studies on the effectiveness of CS in patients with dementia vary from findings that CS has a strong positive effect on cognitive functioning to those concluding that the effects are only marginal^15–17^. Other studies have shown that NPIs provide benefits to PWD and that they are probably cost-effective interventions^18–20^. Several studies highlight the importance of combining PIs and NPIs as the ideal methodology to obtain the best results during dementia^11^.

In this study, we investigated the costs and utility of NPIs in AD, evaluating both the costs and health gains associated with CS interventions in patients with AD.

## METHODS

This study received a positive opinion from the Ethics Committee of the hospital where the research was conducted (Opinion No. 10/2022, approved by the Ethics Committee of Hospital Magalhães Lemos on December 14, 2022). Ethical principles were followed during the entire study period.

All NPI (CS therapy [CST]) sessions allowed the development of ways to deal with cognitive deficits and increase the sociability of PWD. In this way, CST also affected other dimensions, such as functional and psycho-affective dimensions, and improved the well-being and QOL of PWDs.

Data were collected from personal records (paper records) and electronic medical records of each patient. A neuropsychological assessment was carried out at two equivalent moments for both groups. In addition to the elements already mentioned, data related to the degree of the severity of dementia and the neuropsychological and functional profile were collected. Regarding costs, the specific direct costs of NPIs were considered.

### Participants

Two samples were constructed with different purposes. The first sample was made up of 40 patients undergoing outpatient NPIs, and the health gains and costs associated with these interventions were evaluated. The second sample constituted the control group (CG), being as similar as possible to the first, also with 40 patients, with the exception that in this group the patients were not subjected to NPIs.

Patients joined CST groups with a maximum of eight participants. The diagnosis of AD was obtained according to the criteria of the DSM-5^21^ and the Alzheimer's Association and the National Institute on Aging^22^. No patient suffered from a serious illness, and all had normal or corrected hearing and vision conditions. The patients knew how to read and write and showed no psychological or behavioral changes.

### Instruments

In this study, patient data required for neuropsychological, functional, social, and QOL assessments were collected. For neuropsychological and functional assessments, data were collected from the following instruments: Clinical Dementia Rating (CDR)^23^, Mini-Mental State Examination (MMSE)^24^, Addenbrooke's Cognitive Examination-Revised (ACE-R)^25^, Barthel Index^26^, and Lawton and Brody Index^27^. Socioeconomic status was evaluated using the Graffar Index^28^.

To assess QOL, the Quality of Life-Alzheimer's Disease Scale (QOL-AD)^29^ was used. The EQ-5D-3L utility values can be easily converted to quality-adjusted life years (QALYs) and can be used to conduct cost-utility analyses in economic assessments^30–32^. QALYs assess the impact of a specific intervention on lifespan and QOL^32^. As QALYs can be calculated for almost all types of treatments, interventions, or procedures, this indicator has a high comparative potential and is widely accepted, as it allows the comparison of health outcomes^33^.

### Design and procedure

A cross-sectional study design was used in this study. Data collected included sociodemographic and clinical data. A researcher collected the data for this study from January to April 2023 from a psychogeriatric service at a hospital located in the northern region of Portugal that serves PWDs.

The cognitive and functional profile of patients diagnosed with dementia was characterized. In addition to comparing the health gains of the two groups, the additional cost of NPIs was evaluated. This study explored the data through descriptive statistics. To infer and validate the results, the two groups were paired according to sociodemographic characteristics, using non-parametric Pearson's χ^2^ and Fisher's exact tests, as well as odds ratio association measures (OR) and 95% confidence interval (CI) for the OR. This procedure made it possible to identify the similarity between the distributions of the two groups, necessary to make them comparable and minimize possible distortions during the neuropsychological, functional, and dementia staging assessments. These evaluations were continued in the "intervention group" (IG) and the "CG" using Wald's test of multivariate logistic regression. All tests used a 0.05 significance level. The tests are one-tailed, as, according to theory, it is expected that risk factors will affect patients who have undergone NPIs more positively than those without NPIs.

## RESULTS

The CG checked the representativeness and comparability criteria. The cases are representative of the target population, according to the inclusion criteria, as well as the control cases have identical distributions to the intervention cases, as shown in Table 1. This table presents the sociodemographic and clinical characteristics of the two samples. There were no significant differences between the two groups, according to the chi-square test and association measures (OR and 95%CI OR), denoting the comparability between the samples. It was also verified that 73.8% of the patients in both groups take anti-dementia medication (Fisher's exact test with p=0.612) and have the same number of years with dementia (Wald's test=3.381, p=0.07). They differ, however, in the number of years of follow-up (Wald's test=5.446, p=0.02), which is significantly higher in the IG than in the CG, which may be related to earlier interventions in the IG.

**Table 1:** Sociodemographic and clinical characteristics.

Sociodemographic and clinical characteristics		AD patients with intervention (n=40)	Control group (n=40)	p-value
Sex (%) (n)				
	Women	80 (32)	72.5 (29)	0.451
	Men	20 (8)	27.5 (11)	
Age (mean) (SD) (min/max)		78.60 (6.508) (65–93)	79.55 (6.457) (67–93)	0.514
Age group – years (%) (n)				
	≤65	2.5 (1)	0 (0)	0.947
	>65–75	25 (10)	27.6 (22)	
	>75–85	65.0 (26)	56.3 (45)	
	>85–95	7.5 (3)	22.5 (8)	
Marital status (%) (n)				
	Married	65 (26)	47.5 (19)	0.295
	Widower	25 (10)	45 (18)	
	Single	5 (2)	2 (5)	
	Divorced	5 (2)	1 (2.5)	
Years of education (mean) (SD) (min/max)		7.03 (4.526) (1–17)	5.73 (4.132) (0–15)	0.184
Education (%) (n)				
	Illiterate	2.5 (1)	2.5 (1)	0.459
	Reading and writing	2.5 (1)	12.5 (5)	
	4 years	55 (22)	65 (26)	
	9 years	12.5 (5)	2.5 (1)	
	11–12 years	10 (4)	2.5 (1)	
	>12 years	17.5 (7)	15 (6)	
Portuguese nationality (%) (n)		100 (40)	100 (40)	-
Profession (%)				
	Retired	100	100	-
	Other	0	0	
Social class (Graffar) (%) (n)				
	I (high)	10 (4)	5 (2)	0.496
	II (medium-high)	10 (4)	10 (4)	
	III (medium)	55 (22)	70 (28)	
	IV (medium-low)	25 (10)	15 (6)	
Number of years with dementia (mean) (SD)		3.97 (0.37)	3.07 (0.28)	0.07
Number of follow-ups (mean) (SD)		5.53 (0.63)	2.87 (0.29)	0.02
Living alone (%) (n)				
	Yes	10 (4)	0 (0)	0.116
	No	90 (36)	100 (40)	
Pharmacological intervention with antidementia (%) (n)				
	Yes	70 (28)	77.5 (31)	0.612
	No	30 (12)	22.5 (9)	

Table 2 presents the results obtained from the two neuropsychological, functional, and dementia staging assessments carried out for the IG and CG. All variables submitted to multivariate logistic regression analysis resulted from the differences between the two assessment moments in the IG and CG (p-test column for differences). At each of the assessment moments, patients receiving NPIs were compared with those in the CG. The Wald's test showed that no significant differences were found between the two groups at both times in the initial cognitive assessment tests. In the remaining assessments, the time factor differentiated them significantly.

**Table 2:** Impact of non-pharmacological interventions – Neuropsychological and functional assessments of patients with dementia undergoing non-pharmacological interventions (intervention group) and controls (control group).

Neuropsychological and functional assessments		First evaluation		Second assessment		p-value*
		Intervention group	Control group	Intervention group	Control group	
Barthel index (mean) (SD)		97.75 (6.97)	90.63 (13.06)	96.25 (8.06)	84.62 (17.40)	0.014
Lawton index (mean) (SD)		18.25 (4.71)	19.33 (5.5)	18.67 (4.74)	22.65 (5.63)	0.028
MMSE (mean) (min–max) (DP)		22.55 (13–29) (3.74)	19.80 (13–27) (3.71)	21.88 (13–29) (4.43)	18.25 (9–25) (3.96)	0.012
ACE-R (mean) (min–max)		64.93 (33–84)	52.62 (30–76)	62.83 (32–82)	52. 57 (32–76)	0.032
Attention/concentration (mean)		14.21	11.38	13.61	13.71	0.594
Memory (mean)		10.25	7.62	9.65	6.85	0.395
Fluency (mean)		4.75	3.11	5.09	2.48	0.244
Language (mean)		21.57	20.51	21.83	21.142	0.399
Visuoconstructive (mean)		13.39	10.95	12.87	10.71	0.051
Clinical dementia rating	CDR=1 (%) (n)	90 (36)	82.5 (33)	90 (36)	57.5 (23)	0.011
	CDR=2 (%) (n)	10 (4)	17.5 (7)	10 (4)	35 (14)	
	CDR=3 (%) (n)	0 (0)	0 (0)	0	7.5 (3)	

Note:

* Wald's test, significant (one-tailed).

According to Table 2, both groups, on average, performed worse in the second assessment than in the first, with the CG suffering a greater decline in the values obtained in the cognitive and functional assessment tests.

### Health gains for people with dementia undergoing non-pharmacological interventions

We found a statistically significant difference between the results of the first assessment and the results of the second assessment, with higher QOL values obtained in the assessment after the intervention, as measured by the QOL-AD (Wald's test=9.679; p=0.002). In contrast, the CG had a lower result in the second QOL assessment compared to the first assessment.

**Figure 1:**
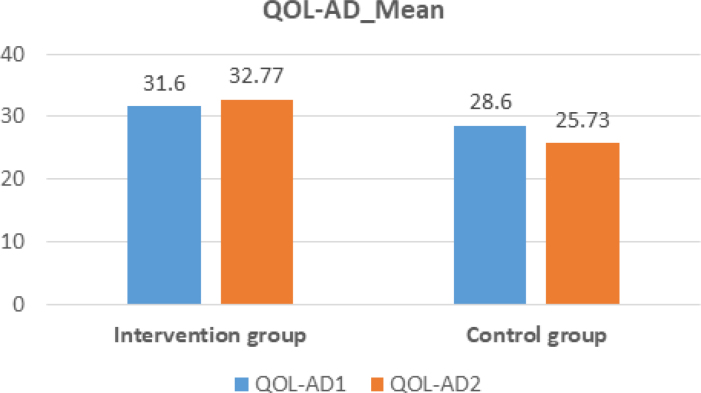
Comparison of the mean result in the QOL-AD assessments between the intervention group and control group.

Figure 1 documents the results obtained in the assessment of QOL with the QOL-AD at the two assessment moments and in the two groups assessed. The IG obtained better results than the CG in the second evaluation, which indicates that the NPI allows health gains to occur.

We also applied the EQ-5D-3L to the two groups evaluated. Economic assessments help improve the efficiency of the health sector by optimally prioritizing, rationing, and allocating resources while also increasing its effectiveness by improving access and equity^34^. According to Hoomans and Severens^35^, economic evaluations apply not only to decisions about interventions or services that directly target patients, such as PIs and medical devices, but also to decisions about implementation strategies, which are explicitly designed to inform care providers and patients about the best available research evidence and to improve its use in their clinical practice.

Table 3 presents the mean relationship of the state of health values obtained through the EQ-5D-3L instrument. This table shows that both groups of patients suffered a deterioration in their health status, with worse results in the second assessment compared to the first assessment. Regarding the comparison of health status in relation to the two groups, assessed with the EQ-5D-3L instrument, we found that in IG, there was a smaller decline in health status.

**Table 3:** Mean relating to health status weights (with the EQ-5D-3L).

Mean value for health status	Intervention group	Control group
First evaluation	0.62833	0.54925
Second evaluation	0.61577	0.39548

Table 4 presents the averages relating to health status weights (with the EQ-5D-3L) by sex, for the two groups. It appears that in both groups there was a deterioration in health status, with worse results in the second assessment compared to the first assessment, except in the group of male patients who participated in the IG and who obtained a better result in the second assessment.

**Table 4:** Mean relating to health status weights (with the EQ-5D-3L) by sex.

Mean value for health status	Intervention group		Control group	
	Women	Men	Women	Men
First evaluation	0.61244	0.69188	0.55759	0.52727
Second evaluation	0.58984	0.71950	0.39072	0.40800
Difference between the first and second evaluations	-0.0226	+0.02762	-0.16687	-0.11927
QALY gains	0.14427	0.14689		

Abbreviation: QALY, quality-adjusted life year.

### Economic evaluation of non-pharmacological interventions

A cost-utility analysis of NPIs in AD, focusing on the costs and health gains associated with CST for PWDs, was performed. Economic analysis helps decision-makers make better choices and use available resources more efficiently^36^. In the economic evaluation, clearly identified and justified assumptions were used, especially regarding the duration and magnitude of the effects of the NPI. The intervention was evaluated in two moments with a period of approximately 9 months (0.75 years), and it was assumed that during this period, only half of the QOL gains assessed after the 9 months of the intervention were achieved. We assume that after 9 months of the intervention, all patients will continue their follow-up, including undergoing day hospital sessions (DHSs) at the same frequency as during the intervention. We also assume that both the costs and the health gains will persist until the time of the patient's death. To estimate the number of years of life remaining after the end of this intervention until the expected time of death, it is necessary to consider the life expectancy at age 65 years.

In Portugal, the life expectancy at 65 years of age in 2020–2022 was estimated at 20.98 years for females and 17.76 years for males^37^. Therefore, it is expected that a female will live until approximately 85.98 years and a male until approximately 82.76 years. On the other hand, the IG submitted to NPIs had an average age for females of 78.84 years and for males of 77.62 years, while the CG had an average age for females of 79. 17 years and for males of 80.55 years. Thus, the estimated number of years of life after 0.75 years for each of the groups will be: IG, females: 7.14–0.75=6.39 and IG, males: 5.14–0.75=4.39.

It was assumed that IG patients would live an average of 6 more years after the intervention (80% women*6.39 years+20% men*4.39 years). This assumption seems reasonable, given that the patients who participated in this study were at an early stage of the disease and there is evidence that shows that people aged 65 years or over survive an average of 4–8 years after being diagnosed with AD, although some live until 20 years with this dementia^1,38–40^, reflecting a slow and insidious progression of AD^1^. Thus, it was considered that after the 9 months of the intervention, the differential between the QOL of the group undergoing the intervention compared to the CG remains the same throughout this 6-year period.

In this study, it was assumed that the participants did not experience an abrupt reduction in their levels of cognition and functionality, but, on the contrary, they suffered a progressive decline in their cognitive and functional abilities, as is the case in AD.

The QALYs gained with the application of the NPI program were given by QALYG=QALYIG–QALYCG, where QALYIG designates the QALYs gained by implementing the health program (CST) in relation to the QALYs obtained without NPIs, despite having carried out other interventions (namely, PIs). Thus, QALYCG designates QALYs obtained with other interventions. For females, the QOL gains estimated with the intervention were 0.14427, while for males, these gains were estimated at 0.14689. Considering the gender composition of the IG, which is 80% of women, a differential gain in QOL per year was estimated as


0.14427*0.8+0.14689*0.2=0.144794.

The costs of NPIs per PWD (a session once a week) for 9 months were calculated knowing that the cost of each DHS in 2022 was €69.39 and that NPIs were carried out in DHSs. When the 9 months ended, there was no abrupt interruption of interventions, and patients continued to undergo NPIs and other usual treatments, namely PIs. The 9 months of NPIs correspond to 39 DHSs (9×52/12=39 sessions). Thus, patients attended 39 sessions, and we obtained the costs per patient, which, for the year 2022, were €2706.21.

The costs and consequences of health interventions do not manifest themselves in a single period but can last for some years, making it necessary to determine the current value of the costs and consequences that will occur in the future.

In accordance with the methodological guidelines for economic evaluation studies, published by the National Authority for Medicines and Health Products (INFARMED)^36^, as there is no official value in Portugal to support investment decisions with public financing, we decided that the costs and consequences should be discounted at an annual rate of 4%. According to Perelman et al.^36^, the discount rate represents the opportunity cost for society to use available resources in the national health service, as they could have been used in other areas of the economy and with a positive rate of return. To ensure comparability, costs and consequences must refer to the same period. By comparing the costs and QALYs obtained with the NPI, it was possible to calculate a cost per QALY. During the 9 months of the intervention, there were hospital costs per person of €2,706.21. In the years after the intervention, there was a QALY differential of 0.144794. After discounting the costs and QALYs, we obtained a discounted cost value per patient of €21,621.31 and QALY gains per patient of 0.81333, which represent a cost per QALY of €26,583.76. Given the decision thresholds usually considered by INFARMED^36^ of €30,000, this intervention is cost-effective from an economic point of view. Table 5 presents the costs and gains with the NPI.

**Table 5:** Costs and consequences (gains) of the non-pharmacological intervention

	Year 0	Year 1	Year 2	Year 3	Year 4	Year 5	Year 6
Costs	2706.21€	3608.28€	3608.28€	3608.28€	3608.28€	3608.28€	3608.28€
Discounted costs	2706.21€	3469.50€	3336.06€	3207.75€	3084.37€	2965.74€	2851.68€
Total discounted costs	21,621.31€						
QALY gains	0.05429	0.14479	0.14479	0.14479	0.14479	0.14479	0.14479
Discounted QALY gains	0.05429	0.13922	0.13387	0.12872	0.12377	0.11901	0.11443
Total discounted QALYs	0.81333						
Costs per QALY	26,583.76€						

Abbreviation: QALY, quality-adjusted life year.

All economic assessments have a degree of uncertainty, imprecision, or controversy regarding some assumptions. In this study, a sensitivity analysis was carried out to assess the extent to which previous results would modify with changes in the values of the assumptions made.

If we opt for a lower discount rate than initially stipulated, keeping the remaining values constant, it could be seen whether the values change significantly. Thus, if we use a discount rate of 3.5%, a value also followed by the NICE^41^, we would estimate a value of 0.82584 total discounted QALYs, with total discounted costs of €21,933.12 and a cost-effectiveness ratio of €26,558.55 per QALY, which shows that the values would have not changed significantly and that the intervention would remain cost-effective.

If we opt for a higher discount rate than initially stipulated, using a discount rate of 5%, keeping other values constant, we would estimate a value of 0.78922 total discounted QALYs (with an annual rate of 5%), with total discounted costs of €21,020.73 and a cost-effectiveness ratio of €26,634.56 per QALY.

If we consider the assumption that costs are 50% higher than those considered in the base scenario, a cost per QALY of €43,605.86 would be obtained, a value higher than that usually considered acceptable by regulatory authorities in Portugal (INFARMED) and several European countries^36^.

If health gains were 20% lower than those considered in the base scenario, maintaining the remaining assumptions, the cost per QALY would be €33,229.70, a value also considered excessive.

## DISCUSSION

Our study provided data that demonstrated the benefits of CS in PWDs, in line with some studies on the effectiveness of CS in PWDs that found that CS indeed had a positive effect on cognitive functioning^12,18–20^. Khan et al.^13^, in another review article, found good evidence supporting the use of CST as an NPI for PWDs. These authors considered that CST showed benefits in cognition and well-being in PWDs in randomized clinical trials. Another study by Chen et al.^12^ confirmed that the combination of CS and drug treatment in AD was effective. Several studies highlight the importance of combining PIs and NPIs as the ideal methodology to obtain the best results during the evolution of dementia^11–13^.

In this study, it was found that CST is a cost-effective NPI. These results are in the same vein as the article by Woods et al.^18^, which showed that several NPIs provided benefits to mild and moderate PWDs beyond the effects of medication and that they were probably cost-effective interventions.

This work also corroborates the investigation carried out by D’Amico et al.^19^, in which it was assessed whether maintenance CST was cost-effective when added to usual care. CST in combination with acetylcholinesterase inhibitors offered cost-effectiveness gains when the outcome was measured as cognition.

The results obtained in our study agree with the results published by Knapp et al.^20^ who investigated the cost-effectiveness of a CST program for PWDs. These authors concluded that CST for PWDs had efficacy advantages and could be more cost-effective than usual care.

One of the limitations of this study is that the results of this economic evaluation were obtained in the context of a psychogeriatric service, which has many specificities and a small sample size, making comparisons with other programs in different health services and their generalization more difficult.

In conclusion, through an NPI of CST implemented in a psychogeriatric service, a cost-effectiveness ratio of €26,583.76 per QALY was obtained. Therefore, we conclude that this intervention is cost-effective.

NPIs, namely CST in the health sector, provide health gains and can also be cost-effective. This supports the recommendation that such interventions, tailored to the needs of patients with AD, should be implemented. In future research, larger samples and/or different contexts should be used to better generalize the results.
